# High Performance Relaxor-Based Ferroelectric Single Crystals for Ultrasonic Transducer Applications

**DOI:** 10.3390/s140813730

**Published:** 2014-07-29

**Authors:** Yan Chen, Kwok-Ho Lam, Dan Zhou, Qingwen Yue, Yanxiong Yu, Jinchuan Wu, Weibao Qiu, Lei Sun, Chao Zhang, Haosu Luo, Helen L. W. Chan, Jiyan Dai

**Affiliations:** 1 The Hong Kong Polytechnic University Shenzhen Research Institute, Shenzhen 518057, China; E-Mails: ap.cheny@connect.polyu.hk (Y.C.); lei.sun@polyu.edu.hk (L.S.); apahlcha@polyu.edu.hk (H.L.W.C.); 2 Department of Electrical Engineering, The Hong Kong Polytechnic University, Hong Kong, China; E-Mail: kokokh.lam@polyu.edu.hk; 3 Edan Instrument, Inc., Shenzhen 518067, China; E-Mails: dannyzhou82@aliyun.com; 4 Information Materials and Devices Research Center, Shanghai Institute of Ceramics, Chinese Academy of Science, Shanghai 201800, China; E-Mails: yue.qingwen@student.sic.ac.cn(Q.Y.); hsluo@mail.sic.ac.cn (H.L.); 5 Shantou Institute of Ultrasonic Instruments Co., Ltd., Shantou 515041, China; E-Mails: yyx@siui.com(Y.Y.); wjc@siui.com(J.W.); 6 Paul C. Lauterbur Research Center for Biomedical Imaging, Institute of Biomedical and Health Engineering, Shenzhen Institutes of Advanced Technology, Chinese Academy of Sciences, Shenzhen 518055, China; E-Mail: wb.qiu@siat.ac.cn; 7 Shenzhen Optomechatronics Key Lab, Research Institute of Tsinghua University in Shenzhen, Shenzhen 518057, China; E-Mail: zhangc@tsinghua-sz.org

**Keywords:** PMN-PT, PIN-PMN-PT, single crystals, composites, ultrasonic transducers

## Abstract

Relaxor-based ferroelectric single crystals Pb(Mg_1/3_Nb_2/3_)O_3_-PbTiO_3_ (PMN-PT) have drawn much attention in the ferroelectric field because of their excellent piezoelectric properties and high electromechanical coupling coefficients (*d*_33_∼2000 pC/N, *k*t∼60%) near the morphotropic phase boundary (MPB). Ternary Pb(In_1/2_Nb_1/2_)O_3_-Pb(Mg_1/3_Nb_2/3_)O_3_-PbTiO_3_ (PIN-PMN-PT) single crystals also possess outstanding performance comparable with PMN-PT single crystals, but have higher phase transition temperatures (rhombohedral to tetragonal *T*_rt_, and tetragonal to cubic *T*_c_) and larger coercive field *E*_c_. Therefore, these relaxor-based single crystals have been extensively employed for ultrasonic transducer applications. In this paper, an overview of our work and perspectives on using PMN-PT and PIN-PMN-PT single crystals for ultrasonic transducer applications is presented. Various types of single-element ultrasonic transducers, including endoscopic transducers, intravascular transducers, high-frequency and high-temperature transducers fabricated using the PMN-PT and PIN-PMN-PT crystals and their 2-2 and 1-3 composites are reported. Besides, the fabrication and characterization of the array transducers, such as phased array, cylindrical shaped linear array, high-temperature linear array, radial endoscopic array, and annular array, are also addressed.

## 1. Introduction

Most humans can hear sound in the 20 Hz to 20 kHz frequency range. The sound waves with frequencies higher than 20 kHz are termed ultrasound. Ultrasound has been used in many fields, including chemistry, nuclear physics, biology, medicine, underwater communication technology, materials testing and so on [1].

Ultrasonic diagnostic techniques, which are effective, very safe, and non-invasive compared to computed tomography (CT), X-ray, and magnetic resonance imaging (MRI), are very important in the medical field. Ultrasound diagnosis is extensively used in cardiology, obstetrics, gynecology, eye scans, blood flow measurement, breast scans, endoscopy, *etc*. [2].

Ultrasonic transducers are typically classified into single element and array transducers, depending on the number of active elements. The array transducers include linear array, radial array, annular array, phased array, and so on. In terms of frequency, ultrasonic transducers can also be categorized into low-frequency, high-frequency (>20 MHz), and ultrahigh frequency (>100 MHz) based on their center frequencies [2].

Recently, high-frequency ultrasonic imaging has been widely used in diagnostics of eyes, skin, and blood vessels, *etc.*, as well as small animal studies [3–5]. In order to more accurately examine diseases in organs such as eye, blood vessels, *etc.*, it is essential to use higher-frequency ultrasonic transducers with broader bandwidth and better resolution. Therefore, one of development trends of medical ultrasonic transducers is to increase the working frequency. In current ultrasonic clinical diagnosis, most of the scanners are large-scale and heavy for multi-functional imaging applications. The patients have to be brought to hospitals for diagnosis. Consequently, development of small and portable ultrasonic equipment is the other trend. In the future, portable ultrasonic equipment, together with other traditional paraphernalia such as the ophthalmoscopes and stethoscopes, will be carried by physicians for patient diagnosis, which will benefit patients greatly [6]. GE developed an I-Pod-sized scanner that has a 88.9 mm isplay and a weight of 390 g [7]. High-quality relaxor-PT single crystals with outstanding piezoelectric performance and relatively high dielectric constant are suitable for miniaturized transducer applications.

As the active element of the transducer, the properties of piezoelectric materials dominate the transducer performance features such as bandwidth and sensitivity. Piezoelectric lead zirconate titanate (Pb(Zr_1−x_Ti_x_)O_3_ (PZT) ceramics have extensively been used as the active element of transducers for applications in both medical diagnostics and nondestructive testing. However, longitudinal piezoelectric constant *d*_33_ and electromechanical coupling coefficient *k*_33_ of PZT are about 500 pC/N and 0.7, respectively [8–10], which still leave much room to further improve the sensitivity and resolution for medical ultrasound imaging systems. Therefore, with the development of the electronic technology and medical ultrasound, it is necessary to investigate new piezoelectric materials to meet the requirements of high-performance ultrasonic transducers with high resolution and broad bandwidth [11–14].

Relaxor-based ferroelectric single crystals (1−x)Pb(Mg_1/3_Nb_2/3_)O_3_-xPbTiO_3_ (PMN-PT) have drawn much attention because of their excellent piezoelectric properties near the morphotropic phase boundary (MPB) composition [15]. The *d*_33_ and *k*_33_ are reported to be >1500 pC/N and ∼0.9, respectively. Besides the binary materials, ternary Pb(In_1/2_Nb_1/2_)O_3_-Pb(Mg_1/3_Nb_2/3_)O_3_-PbTiO_3_ (PIN-PMN-PT) single crystals also possess outstanding piezoelectric properties and even higher Curie temperatures compared to the PMN-PT single crystals [16]. Therefore, these relaxor-based PMN-PT and PIN-PMN-PT single crystals have potential applications for ultrasonic transducer applications.

Based on the outstanding performance of relaxor-based single crystals, devices with superior properties have been developed, such as electromechanical devices [17–19], piezoelectric transformers [20], and ultrasonic transducers [21–24]. Besides, these single crystals have also been used as promising candidates for medical imaging applications [23,25,26]. Philips Co.'s “pure-Wave” technology started using the PMN-PT single crystal for commercial medical imaging transducer application [26,27]. Shung's group reported the use of a high-frequency PMN-PT ultrasonic needle transducer for pulsed-wave Doppler applications [23]. Besides, a PMN-PT single crystal high frequency kerfless phased array was also studied [28]. Yamamoto *et al.* investigated the repoling effect on the properties of the PMN-PT single crystal silver form array transducers [29]. Vibration mode and relevant ultrasonic applications of the PMN-PT single crystal were investigated by Luo's group [30]. Therefore, the third development trend is to develop commercial transducers based on high-performance ferroelectric single crystals. In this article, various types of ultrasonic transducers we have fabricated using PMN-PT and PIN-PMN-PT single crystals are reviewed and summarized.

## 2. Relaxor-PT Ferroelectric Single Crystals

### 2.1. PMN-PT Single Crystal

**Figure 1.**
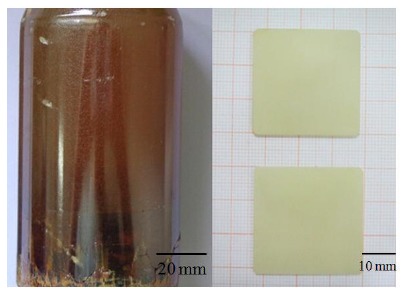
Large-size and high-quality PMN-PT single crystal grown at the Shanghai Institute of Ceramics by the modified Bridgman technique.

PMN-PT single crystals were grown by Shrout *et al.* using a flux growth technique in 1990 [31]. Yamashita, Saitoh *et al.* introduced the relaxor-based ferroelectrics for ultrasonic transducer applications in 1994 [32–34]. Since then, relaxor-based PMN-PT ferroelectric single crystals have drawn more attention [31]. Figure 1 shows the large-size and high-quality PMN-PT single crystal grown by Luo *et al*. from the Shanghai Institute of Ceramics of the Chinese Academy of Sciences using a modified Bridgman technique.

**Figure 2.**
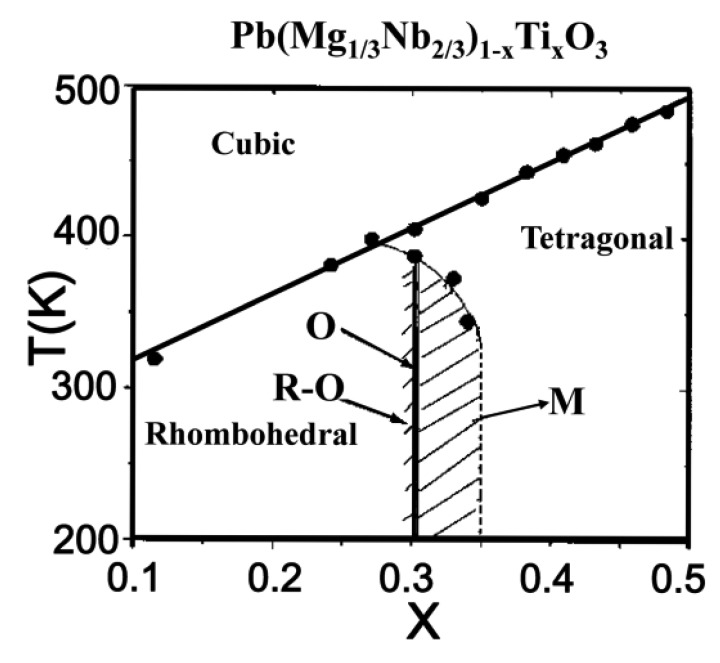
Phase diagram of the PMN-xPT binary system (with permission from [35]).

PMN-xPT single crystals exhibit ultrahigh piezoelectric properties at the compositions near the MPB. Figure 2 shows a complex phase diagram of PMN-PT binary system with rhombohedral (R), tetragonal (T), orthorhombic (O), and monoclinic (M) phases. It can be seen that the MPB composition has PT content, x = 0.28 – 0.36. When the PT content is less than 0.28, the rhombohedral ferroelectric phase is dominated for the PMN-xPT single crystals below the permittivity peak temperature (T_m_). When the PT content is larger than 0.36, the tetragonal ferroelectric phase appears and behaves as a normal ferroelectric [35,36].

### 2.2. PIN-PMN-PT Single Crystal

Recently, a ternary relaxor ferroelectric single crystal, PIN-PMN-PT has been grown successfully by the modified Bridgman method [37–39]. The ternary PIN-PMN-PT single crystals possess similar ultrahigh piezoelectric and electromechanical properties like binary PMN-PT single crystals, but the ternary ones have higher phase transition temperatures (rhombohedral to tetragonal *T*_rt_ and tetragonal to cubic *T*_c_) and larger coercive field *E*_c_ than those of the binary ones [25,40–42]. This widens the applications of relaxor-based ferroelectric single crystals under high temperature and high power conditions [43,44]. With the enhanced thermal and electrical stability, the use of PIN-PMN-PT single crystals for ultrasonic transducer applications has been reported [25,43].

**Figure 3.**
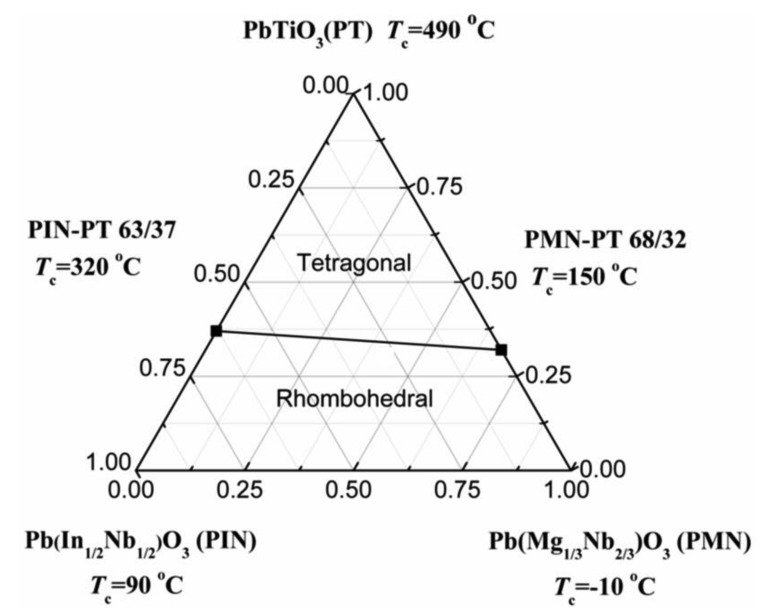
Phase diagram of PIN-PMN-PT ternary system (with permission from [45]).

Figure 3 shows the phase diagram of the ternary PIN-PMN-PT system. It can be found that the PIN-PMN-PT crystals exhibit the tetragonal phase above the MPB line, and the rhombohedral phase below the MPB line.

### 2.3. Properties of Relaxor-PT Single Crystals

Relaxor-PT-based ferroelectric single crystals near the morphotropic phase boundary (MPB) have been widely investigated during the past decades due to their significantly high electromechanical coupling coefficients (*k*_t_ ∼60%), extremely large strains (>1%) and extremely high piezoelectric coefficients (*d*_33_>2000 pC/N) compared with PZT-based piezoceramics (strain ∼0.1%, *k*_t_∼50%, *d*_33_∼400–600 pC/N). Cao's group studied the domain engineering and characterization of the relaxor-based ferroelectric single crystals to understand their giant piezoelectric properties [46]. The Zhang group reported the recent development of the relaxor-PT single crystals for various electromechanical applications [47–49]. Table 1 lists some of the important piezoelectric properties of PZT-5H ceramic, PMN-PT, and PIN-PMN-PT single crystals.

## 3. Single-Element Ultrasonic Transducers

### 3.1. Single-Element Endoscopic Ultrasonic Transducer

Endoscopic ultrasound (EUS) is a medical procedure in endoscopy combined with ultrasound in order to obtain images and information of the digestive tract and the surrounding tissues and organs. In EUS imaging, a miniaturized ultrasonic transducer is installed on the tip of the endoscope. By inserting the endoscope into the digestive tract via the mouth, ultrasonic images of the organs or tissues inside the body can be obtained [53–60]. Compared with traditional ultrasound, EUS imaging is more accurate, and gives more detailed information due to the proximity of the EUS transducer to the organs of interest. This methodology proves to be effective, safe, minimally-invasive, and very well tolerated, which has been investigated by numerous studies [53,61–63]. However, the currently used endoscopic-type ultrasonic transducers are mainly made from piezoelectric PZT ceramics, whose properties are not as good as those of PMN-PT single crystals. In this section, the single-element EUS transducers using PMN-PT single crystals will be reported. Besides, the radial array EUS transducer will also be discussed in the part of array transducer (Section 4).

**Figure 4.**
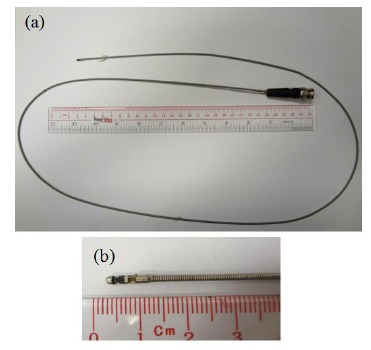
The prototype of a 10 MHz rotatable ultrasonic (**a**) catheter and (**b**) enlarged probe [64].

Figure 4 shows the prototype of a 10 MHz rotatable ultrasonic imaging catheter. The probes are made of stainless steel, and the transducer element is attached in the trough near the tip. The transducer is attached to the flexible metal tube by gluing or welding. The diameter and length of the transducer metal housing are about 1.8 and 7.0 mm, respectively. The endoscopic transducer probes are fabricated using the PMN-PT single crystal. The frequency of the transducers is 10 MHz. The length of the flexible metal catheter is around 1.5 m long. The other end of the catheter is connected with a Bayonet Neill-Concelman (BNC) connector. The size of the piezoelectric element is 2.5 mm × 1.0 mm with the thickness of ∼200 μm for the 10 MHz transducer. The single-element EUS probe includes a flexible shaft driven by a motor so as to rotate the transducer mechanically, forming a 360-degree image.

**Figure 5.**
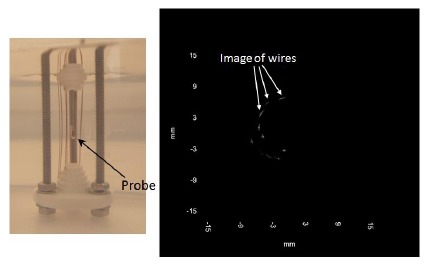
Photograph of the wire phantom and wire phantom image captured by the 10 MHz endoscopic transducer.

The bandwidth was determined to be 62%. The corresponding insertion loss was measured and calculated to be 24 dB. Figure 5 shows the photograph of the wire phantom and wire phantom image captured by the 10 MHz endoscopic transducer. It can be seen that the copper wires are placed around the probe. When the transducer rotates, the wire phantom image can be captured clearly [64].

### 3.2. Intravascular Ultrasonic Transducer

Atherosclerosis is one of the primary causes of human morbidity and mortality due to the formation of plaques in the arteries. Recently, intravascular ultrasonic (IVUS) transducers are increasingly used for the diagnosis of cardiovascular diseases, such as atherosclerosis. The cross-sectional ultrasonic images of the vessel wall can be captured by a thin catheter with a miniaturized ultrasonic transducer placed at the tip of the end of the catheter [65]. Commercial IVUS transducers are mostly fabricated using PZT ceramics, whose properties are stable, but not as good as the recently developed PMN-PT single crystals which have been demonstrated to be the best piezoelectric material (where x = 0.28 − 0.30) so far. Besides, the IVUS transducers are also very expensive and suggested for single usage [6]. Most of the commercialized IVUS transducers are produced by the Volcano Corporation [66] and Boston Scientific [67]. Moreover, a 50 ohm-impedance matching may be easily achieved with a reduced device size for the PMN-PT based transducer due to its high dielectric constant compared to that of LiNbO_3_, PVDF and ZnO materials. This makes it possible to realize miniaturized transducers without degradation in performance, which is particularly interesting for intravascular applications.

**Figure 6.**
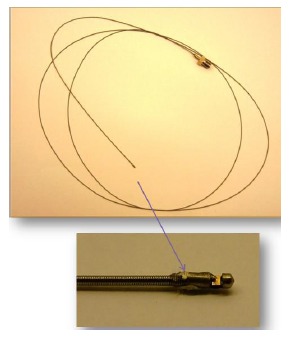
The IVUS transducer based on PMN-PT single crystal [64].

Figure 6 shows an IVUS transducer based on PMN-PT single crystal. The active element size is 0.6 mm × 0.6 mm, and the diameter of the catheter is about 0.7 mm. The backing and matching layers of the IVUS transducer are silver epoxy (E-solder 3022) mixed with tungsten powder, and parylene C, respectively. The other end of the catheter is connected with a SubMiniature version A (SMA) connector. The pulse-echo waveforms and frequency spectra of the PMN-PT IVUS transducers have been tested. The center frequency of the transducer is in the range of 32 to 40 MHz, and their −6 dB bandwidth is about 60%. The insertion loss of the IVUS transducers approaches 25 dB.

**Figure 7.**
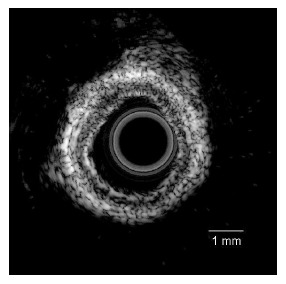
Ultrasonic image of the swine coronary artery captured by the IVUS transducer (with permission from [65]).

**Figure 8.**
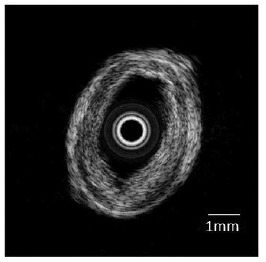
Ultrasonic image of human coronary artery captured by the IVUS transducer [64].

Figure 7 shows the ultrasonic image of the swine coronary artery imaged using the PMN-PT single crystal IVUS transducer. By inserting the transducer into the arteries, the cross-sectional ultrasonic images of the artery can be clearly observed. Besides, it is apparent that different layers of the artery can be well distinguished in the ultrasonic image. Figure 8 shows the ultrasonic image of the human coronary artery. The image possesses high signal-to-noise (SNR) ratio and good contrast [64].

### 3.3. High-Frequency Ultrasonic Transducer

High-frequency ultrasonic transducers have been used widely for imaging small animals, eyes, and arteries [2–4]. Adding a lens and shaping the piezoelectric element into a focused surface are the common ways of fabricating focusing transducers. Among the focusing transducer fabrication methods, the shaped element used in transducers was reported to be much effective for fabricating high-sensitivity devices [68,69]. Hard pressing and pressure defection techniques are the usual ways to shape transducer elements [70–72]. For flexible composite and polymer materials, focusing transducers can be easily fabricated using those techniques. Nevertheless, short-circuit and degradation may occur during hard pressing the bulk ceramic and single crystal elements. Previously, a mechanical dimpling technique was reported to be an alternative way to fabricate high-resolution focusing transducers [73].

#### 3.3.1. High-Frequency Focusing PMN-PT Ultrasonic Transducer by a Dimpling Technique

High-frequency focusing transducers were developed by a dimpling technique [74]. The [001]-oriented PMN-0.28PT single crystal was used as the active element of the ultrasonic transducers. For performance comparison, the plane ultrasonic transducers with similar configurations as the focusing transducers were also fabricated.

As a focusing transducer element, the front-face of the single crystal was dimpled using a dimple grinder (Gatan, Model 656). The focal length of the sample depends on the radius of the grinding wheel. After grinding to the designated depth, the curved surface was polished using a diamond lapping paste wheel with the same radius. Then, the single crystal was diced to a proper dimension using a laser micro-machining technique.

**Figure 9.**
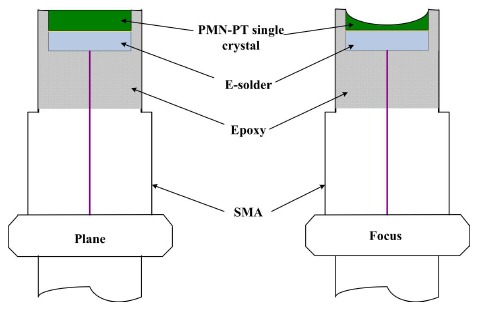
A schematic diagram of the plane and focusing transducers (with permission from [74]).

**Figure 10.**
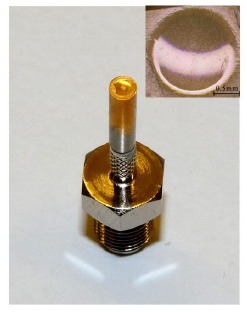
Photographs of the 27 MHz focusing transducer and dimpled element (inset) (with permission from [74]).

To compare the performance with the dimpled focusing transducers, plane transducers were also fabricated. Figure 9 shows schematic diagrams of the plane and focusing transducers. The entire configuration is almost the same for both the plane and focusing transducers. There is no matching layer for both plane and focusing transducers for comparison. A SMA connector was assembled as an electrical connection. Figure 10 shows the photographs of the 27 MHz focusing transducer and dimpled element (inset). It is seen that the appearance of the dimple is obviously round in shape. The concave surface is smooth, without the existence of any crack, indicating that the mechanical dimpling technique can produce high-quality spherical shape even on the brittle crystal elements.

Table 2 summarizes the performances the plane and focusing PMN-PT transducers. Because of the reduction of the crystal thickness after dimpling, the center frequency of the focusing transducer is higher than that of the plane one. Considering multi-resonances from a continuous change of thickness along the curve surface of the dimpled crystal, the dimpled focusing transducer exhibits broader bandwidth and better sensitivity than the plane one [73]. Besides having enhanced performance compared to the plane transducers, the dimpled transducers are also found to be comparable to a hard-press focusing transducer [23]. These results suggest that the mechanical dimpling technique can be used to fabricate high-frequency focusing transducers with broader bandwidth and higher sensitivity.

**Figure 11.**
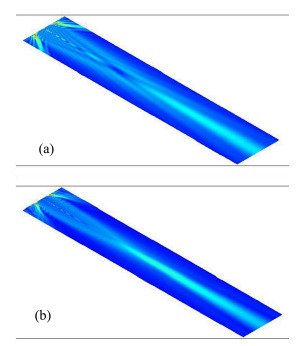
Pressure fields of the (**a**) plane (20 MHz) and (**b**) focusing (27 MHz) PMN-PT transducers [64].

In order to better understand the difference between the plane and focusing transducers, pressure field simulations were carried out. Figure 11 show the pressure field of the plane (20 MHz) and focusing (27 MHz) PMN-PT transducers, respectively. The pressure fields for both transducers are simulated using the PZFlex software (Weidlinger Associates Inc. New York, NY, USA). It can be seen that the pressure field at the focal point of the focusing transducer is stronger and concentrates in a smaller region. In addition, the beam for the focusing transducer is relatively narrower in the far field compared to the plane transducer. The results of the pressure field simulation also illustrate the good sensitivity and better resolution [64].

The dimpled focusing transducers show promising results, indicating that the mechanical dimpling technique can be used to fabricate a high-performance focusing transducers even if the active elements are brittle materials.

#### 3.3.2. High-Frequency PIN-PMN-PT Ultrasonic Transducer

The piezoelectric properties of the PIN-PMN-PT single crystal are comparable to those of the PMN-PT single crystal, but PIN-PMN-PT has higher *T*_rt_ and Curie temperature *T*_m_ and lower clamped dielectric constant (ε_r_ = 713) and acoustic impedance (Z = 34 MRayl) than those of PMN-PT single crystal (ε_r_ = 1032, Z = 37 MRayl) [51,75]. The relatively higher *T*_m_ and lower clamped dielectric constant of PIN-PMN-PT would increase the feasibility of the device design and fabrication. With higher phase transition temperatures, PIN-PMN-PT transducer can operate properly in a broader temperature range. Therefore, compared with the PMN-PT single crystal, the PIN-PMN-PT single crystal has obvious superiority especially in high-frequency and high temperature ultrasonic transducer applications [25,76].

A 40 μm-thick PIN-PMN-PT single crystal with an area of 0.6 × 0.6 mm^2^ was used as the active element of the transducer. Acting as a matching and protective layer, a ∼10 μm-thick parylene C was evaporated onto the front-face surface of the transducers. A conductive epoxy (E-solder 3022, supplied by Von Roll Isola, New Haven, CT, USA) was used as the backing material of the transducer. The center frequency of the transducer is found to be ∼57 MHz. The PIN-PMN-PT transducer exhibits a −6 dB bandwidth of 73%. The insertion loss of the transducer is 20 dB at the centre frequency. The transducer performance is shown to be good for imaging applications.

**Figure 12.**
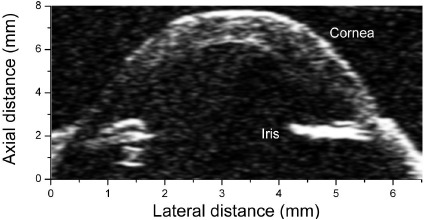
An image of a fish eye captured by the PIN-PMN-PT transducer (with permission from [75]).

Figure 12 shows the image of a fish eye captured using the PIN-PMN-PT transducer, where the anterior portion of the fish eye can be seen and the cornea and iris are well distinguished. Table 3 summaries the performance of PIN-PMN-PT transducers at room temperature. Compared to previously reported results, the resolutions have been enhanced with increased center frequency of the transducer [25].

### 3.4. PIN-PMN-PT Single Crystal/epoxy Composite for Ultrasonic Transducer

#### 3.4.1. PIN-PMN-PT Single Crystal/epoxy 1-3 Composite for High Temperature Ultrasonic Transducer

Piezoelectric composites have been extensively investigated due to their better properties than single phase materials. There are ten connectivity patterns according to different connectivities between the piezoelectric element and the polymer phase [77]. The performance of the 1-3 composite configuration is much more suitable for broad bandwidth ultrasonic transducer applications due to several advantages. First of all, the biphasic 1-3 composite utilizes a high longitudinal mode *k*_33_ instead of a conventional thickness mode *k*_t_ which improves the efficient conversion between electrical and mechanical energy as compared with the single phase material. Secondly, the 1-3 composite can provide lower acoustic impedance *Z* compared to the bulk element which is beneficial for broad bandwidth transducers design and fabrication, since the acoustic impedance of human tissues (∼1.5 MRayl) is much lower than that of the single crystals (≥30 MRayl). Thirdly, the 1-3 composite overcomes the brittleness defects of the single crystal. Fourthly, the electrical properties of the 1-3 composite can be adjusted by changing the volume fraction of the piezoelectric phase which would meet different transducer applications [78].

In this section, a 2 MHz single element ultrasonic transducer was designed and fabricated based on PIN-PMN-PT/epoxy 1-3 composite with 0.5 volume fraction of PIN-PMN-PT single crystal. For the purpose of high-temperature application, the transducer was characterized under different temperatures.

The acoustic impedance of the 1-3 composite is 15 MRayl, which is lower than that of the PIN-PMN-PT single crystal (34 MRayl). Furthermore, the electromechanical coupling coefficient *k*_t_ of the composite is also improved to a higher value of 0.75 compared to that of the single crystal (0.56). Therefore, the transducer performance could be further enhanced using this 1-3 composite.

**Figure 13.**
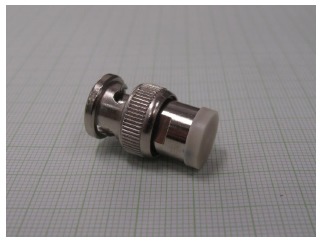
Photograph of the 1-3 composite single-element ultrasonic transducer (with permission from [79]).

For high temperature applications, besides the transducer element, all the other components of the transducer are also designed to be capable to withstand high temperature even after the Curie point of the 1-3 composite. Figure 13 shows the photograph of the single element 1-3 composite ultrasonic transducer.

**Figure 14.**
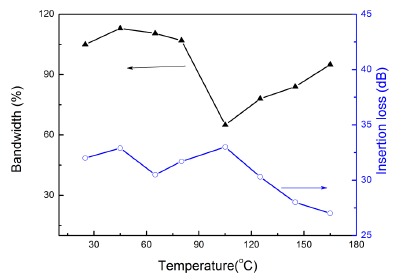
Temperature dependence of the bandwidth and insertion loss of the 1-3 composite single element ultrasonic transducer (with permission from [79]).

The pulse-echo performance and insertion loss of the transducer was measured in a silicone oil bath in front of a stainless steel block at different oil temperatures. Figure 14 shows the temperature dependence of bandwidth and insertion loss of the 1-3 composite transducer. It can be seen that the bandwidth stays at a large value (>100%) and remains almost unchanged from 25 °C to 80 °C. With further temperature increase, the bandwidth decreases to 65% at 100 °C which may be due to the phase transition from rhombohedral to tetragonal. As the temperature increases to 165 °C, the bandwidth of the transducer increases to 95%. The transducer retains its broad bandwidth and good insertion loss at 165 °C, suggesting that the PIN-PMN-PT single crystal/epoxy 1-3 composite has great potential for high temperature ultrasonic applications [79].

#### 3.4.2. PIN-PMN-PT Single Crystal/epoxy 2-2 Composite and 1-3 Composite for 2 MHz Ultrasonic Transducer

**Figure 15.**
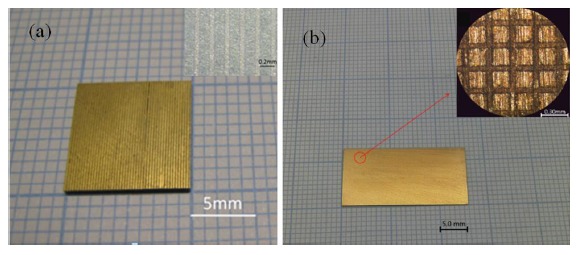
(**a**) Photograph of the PIN-PMN-PT/epoxy 2-2 composite and enlarged image of a randomly selected area on the composite (inset); (**b**) Photograph of the PIN-PMN-PT/epoxy 1-3 composite and enlarged image of a randomly selected area on the composite (inset) (with permission from [80,81]).

Two different composites were designed and developed for 2 MHz ultrasonic transducers. PIN-PMN-PT/epoxy 2-2 composite was designed with 0.7 PIN-PMN-PT volume fraction and the 1-3 composite was designed with 0.59 volume fraction based on the consideration of different piezoelectric modes. The photographs of two composites and their enlarged images are shown in Figure 15. Although the 2-2 composite has similar piezoelectric perfomance as the 1-3 composite, it is much easier to fabricate because of the configruation. Table 4 shows the properties of the PIN-PMN-PT single crystal and its corresponding 2-2 and 1-3 composites. It has been found that the *k*_t_ values of the composites are much higher than those of the single crystal.

Ultrasonic transducers were fabricated based on the PIN-PMN-PT single crystal, 2-2 composite, and 1-3 composite. The center frequency of the transducers were 2.06 MHz, 2.11 MHz, and 1.95 MHz, respectively. The corresponding −6 dB bandwidths were found to be 70.9%, 93.1%, and 94.6%, respectively. The transducer performance is summarized in Table 5. It is seen that the transducers fabricated using the composites not only exhibit broader bandwidth, but also higher sensitivity compared with the single crystal transducer.

## 4. Ultrasonic Arrays

### 4.1. Phased Array

A phased array usually consists of multiple individual elements packed in a housing. All individual elements are generated simultaneously to emit an ultrasound beam, which can be focused and controlled electronically without moving the phased array by using time delays in the electrical generation of each element [82]. It is well known that the ultrasonic arrays with broad bandwidth are important for high-resolution ultrasound imaging. In order to get high resolution and good sensitivity ultrasonic images, PMN-PT single crystals with high performance were used for the phased array fabrication.

#### 4.1.1. 16-Element Phased Array

**Figure 16.**
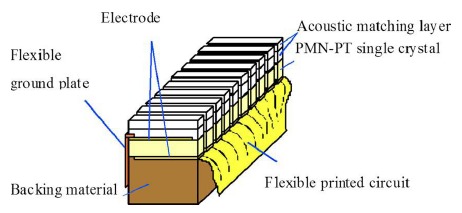
Schematic diagram of the PMN-PT phased array (with permission from [24]).

Figure 16 shows the schematic diagram of the PMN-PT phased array. It can be seen that the phased array is composed of backing material, double matching layers, PMN-PT single crystal, and a flexible printed circuit. A 4.0 MHz PMN-PT transducer array with 16 elements was fabricated and characterized in a pulse-echo arrangement. A hard backing with acoustic impedance of 16 MRayl was used for the 16-element phased transducer. Double 1/8-matching scheme was introduced for improving the transducer performance [24].

**Figure 17.**
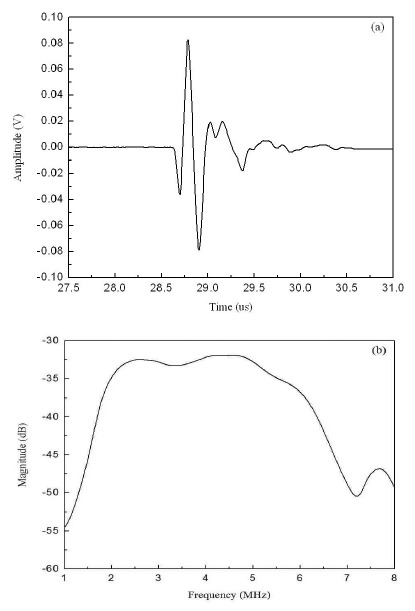
(**a**) Pulse-echo response and (**b**) frequency spectrum of the phased array (with permission from [24]).

The pulse-echo response and frequency spectrum of the phased array are shown in Figure 17. The center frequency and bandwidth of the array element are 4 MHz and 110%, respectively. The insertion loss of the phased array element is about 46.5 dB at the center frequency. The 16-element phased array transducer with hard backing and double 1/8 matching layers was shown to exhibit broader bandwidth (over 100%) than that of the PZT commercial transducer (70%) [24].

#### 4.1.2. 80-Element Phased Array

**Figure 18.**
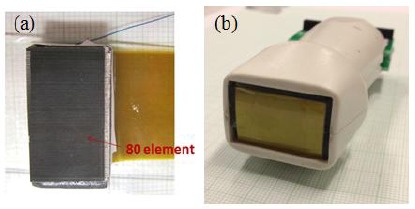
Photographs of the 80-element phased array (**a**) before and (**b**) after adding a lens and housing.

In order to further investigate the phased array transducer, an 80-element phased array was designed and fabricated using PMN-PT single crystals. Figure 18 shows the photograph of the phased array before and after adding a lens (focal length: 80 mm) and housing. It can be found that the 80 elements are uniform and detachment between the matching layers and the single crystal is not found as seen in Figure 18a. The flexible printed circuit was used to connect the array element with a printed circuit board for measurement. The backing layer with a thickness of 10 mm was prepared by mixing tungsten powder, micro bubbles and epoxy 301. The acoustic impedance of the backing layer is 6.5 MRayl. Double matching layers with a thickness of a quarter of wavelength were adopted.

After adding a lens, the phased array transducer presented a broad bandwidth of 92% at the center frequency of 3 MHz. The insertion loss of the phased array was determined to be ∼60 dB. The results show that PMN-PT single crystal has good potential for broad bandwidth phased array transducer applications.

### 4.2. Linear Array

#### 4.2.1. Cylindrically Shaped Linear Array Using PIN-PMN-PT/epoxy 1-3 Composite

**Figure 19.**
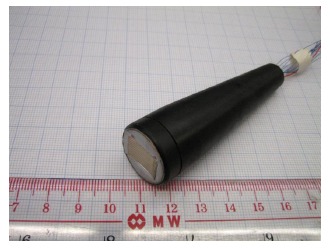
Photography of as-fabricated cylindrically shaped ultrasonic linear array.

**Figure 20.**
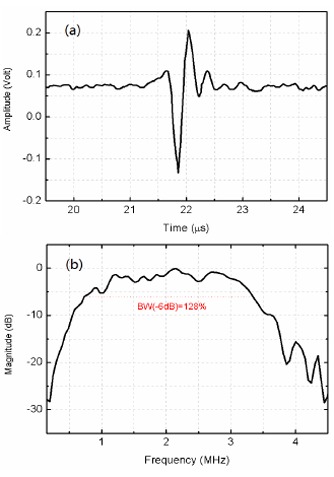
Pulse echo response and frequency spectrum of the cylindrically shaped linear array (with permission from [81]).

Based on the improved flexibility of as-prepared PIN-PMN-PT/epoxy 1-3 composite, a cylindrically shaped ultrasonic linear array has been designed, fabricated and characterized. The cylindrically shaped linear array was found to achieve an ultra-broad bandwidth (−6 dB) of 128%, which is a significant improvement over the 16-element 5.2 MHz PZT linear array produced by Olympus (BW = 70%) [83]. A photograph of the as-fabricated cylindrically shaped linear array with plastic housing is shown in Figure 19. Its pulse echo response and frequency spectrum are shown in Figure 20. The promising results show that the PIN-PMN-PT/epoxy 1-3 composite can be used to develop high-performance ultrasonic linear arrays [81].

#### 4.2.2. PMN-PT/PIN-PMN-PT Linear Array and the Thermal-Independent Properties

With the enhanced thermal and electrical stability, PIN-PMN-PT single crystals have been reported for single-element transducer applications. Actually, compared to the single-element transducer, the array systems are much more desirable because the ultrasonic beam can be dynamically steered and focused in the image plane with high frame rates using electronic scanning. 32-element 3 MHz linear array transducers have been designed and fabricated using both the PMN-PT and PIN-PMN-PT single crystals [76]. The echo waveforms and frequency spectra of the PMN-PT/PIN-PMN-PT single-crystal linear-array ultrasonic transducer were measured at elevated temperatures. Table 6 summarizes the PMN-PT/PIN-PMN-PT single crystal array transducer performance as a function of temperature.

Although the PMN-PT array transducer exhibits a better insertion loss parameter up to 140 °C, compared with the PIN-PMN-PT array transducer, the insertion loss was found to increase at higher temperature due to the low phase transition temperature of the PMN-PT single crystal. However, the performance of the PIN-PMN-PT array was found to remain almost unchanged from room temperature to 160 °C. These results obviously indicate that the PIN-PMN-PT single-crystal array shows good thermal stability and great potential for high-temperature ultrasonic array applications [76].

### 4.3. Radial Array Endoscopic Ultrasonic Transducer

**Figure 21.**
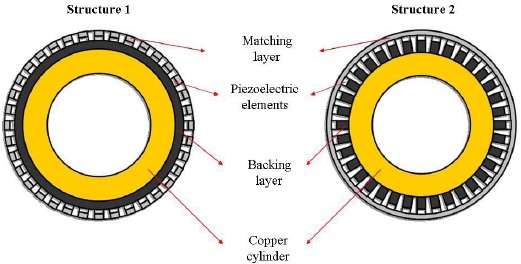
Cross-sectional schematic diagrams of PMN-PT endoscopic ultrasonic radial arrays (with permission from [85]).

**Figure 22.**
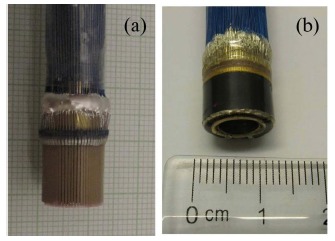
Photographs of the radial array transducers with (**a**) PMN-PT/epoxy 1-3 composite (Structure 1); and (**b**) PMN-PT single crystal (Structure 2) (with permission from [85]).

Radial arrays consist of multiple strip array elements arranged neatly parallel to the cylinder axis. The radial array transducer is used for endoscopy to acquire the images and information of the digestive tract. Compared to the single element endoscopic ultrasonic transducer, the ultrasound images of the organs and tissues acquired by the radial array transducer would be faster and clearer without involving the mechanical rotation. Recently, a rotate-and-dice method was reported for the radial array transducer fabrication based on PZT tubes [84]. However, for the single crystal, it is difficult to fabricate a uniform tube with optimized performance due to different orientations of the crystal, a wrapping method was adopted for the PMN-PT single crystal radial array transducer. The cross-sectional schematic diagrams of PMN-PT endoscopic ultrasonic radial arrays are presented in Figure 21. Two different structures were obtained by using different fabrication procedures. The two structures of the radial array transducers are cutting off the matching layer and the backing layer, respectively. The radial array transducers had the same main components including the piezoelectric layer, backing layer, matching layers, and metal cylinder. The copper cylinder was used for supporting the radial array elements and connecting with the ground electrodes of the elements [53]. Photographs of radial array transducers with different structures fabricated using PMN-PT/ epoxy 1-3 composite and PMN-PT single crystal are shown in Figure 22a and b.

The center frequency and bandwidth of the 64-element 1-3 composite array were found to be 6.9 MHz and 102%, respectively. The performance of the 1-3 composite array is better than that of the 128-element PMN-PT radial array which exhibits a bandwidth of 78% at the center frequency of 3.9 MHz. The two structures of the radial arrays show much larger bandwidth than the commercial PZT array (∼70%). The results suggest that the PMN-PT single crystal show high potential for exhibiting broader bandwidth for endoscopic radial array applications [53,85].

### 4.4. High-Frequency Annular Array

**Figure 23.**
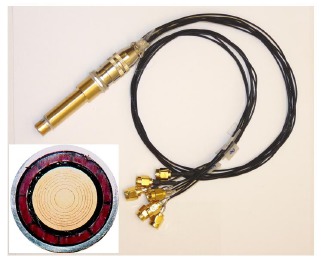
Photograph of the annular array transducer [88].

An annular array is composed of a serial of concentric piezoelectric rings with certain area for emitting at different focal lengths. For the annular array, multi-focal dynamic points with deeper focal length can be operated compared to the single element transducer. Besides, an axisymmetric beam generated from the annular array element is different from that of the linear array. Previously, different materials were employed to develop annular arrays. Snook *et al.* developed a 45 MHz annular array with 6 elements fabricated using PbTiO_3_ ceramic [86]. The other 40 MHz annular array was designed by Ketterling *et al.* based on the polyvinylidene fluoride (PVDF) film [87]. In this section, the PMN-PT single crystal was selected for the annular array design due to its excellent properties. Figure 23 presents the photograph of the 8-element PMN-PT annular array transducer [88]. The element in the annular array was cut off and separated using the 355 nm Nd:YAG laser equipment [89]. The kerf of the annular array was filled using epoxy after cleaning. Wire-bonding technique was used to weld a PCB board with each element for the electrical cable connection [88].

Table 7 summarizes the properties of individual element in the PMN-PT annular array. The bandwidth of the 8-element annular array is in the range of 41% to 78% at about 35 MHz. The insertion loss of the element is good in the range of 18 dB to 28 dB. The results indicate the PMN-PT single crystal is promising for the annular array application.

## 5. Conclusions

Recent developments in the field of single-element and array transducers based on PMN-PT, PIN-PMN-PT single crystals and their 1-3 composites have been reviewed and summarized. Single-element endoscopic and IVUS ultrasonic imaging catheters with good performance based on PMN-PT single crystals were described. Besides, a mechanical dimpling technique was introduced to develop high-frequency focusing ultrasonic transducers, which shows that this novel technique could be the alternative way to fabricate high-frequency focusing transducer for medical imaging applications. For the PIN-PMN-PT single crystals and their 2-2 and 1-3 composites, with promising piezoelectric performance, ultrasonic transducers were developed for high frequency and high temperature studies. Furthermore, phased array, linear array and endoscopic radial array with broad bandwidth have been successfully developed. Finally, the 8-element high frequency annular array with low insertion loss was developed using the PMN-PT single crystal. All the results illustrate that relaxor-based PMN-PT and PIN-PMN-PT ferroelectric single crystals and their composites have great potential in the medical ultrasonic transducer applications. With mature development of single crystals, it is expected that these single crystals with excellent properties will be used more extensively in ultrasonic devices in the near future.

## Figures and Tables

**Table 1.** Piezoelectric properties of PZT-5H ceramic, [001]-poled PMN-PT and PIN-PMN-PT single crystals.

	**PZT-5H ceramic [48,50]**	**PMN-0.28PT SC [51]**	**PIN-0.40PMN-0.33PT SC [52]**
*d*_33_ (pC/N)	593	2365	2742
*d*_15_ (pC/N)	741	132	232
*d*_31_ (pC/N)	−274	−1283	−1337
*k*_33_	75%	94%	95%
*k*_15_	68%	28%	20%
*k*_31_	39%	76%	65%
*k*_t_	51%	63%	59%
*ε*^T^_33_/*ε_0_*	3400	6833	7244
*ε*^S^_33_/*ε_0_*	1470	1032	659
*E*c (kV/cm)	7.0	2.0	5.5
*Tm (°C)*	193	133	197

**Table 2.** Summary of the plane and focusing PMN-PT transducer performance [64].

**Transducer Type**	***f*_c_ (MHz)**	**BW (%)**	**IL (dB)**
Plane	20	34	22
	63	42	28
Dimpled focusing	27	75	11
	77	77	24
Hard-press focusing [23]	44	45	15

**Table 3.** Summary of the performance of PIN-PMN-PT transducers at room temperature [64].

	***f*_c_ (MHz)**	**BW (%)**	**IL (dB)**	**Axial Resolution (μm)**	**Lateral Resolution (μm)**
**PIN-PMN-PT**	57	73	20	26	127
**PIN-PMN-PT**[25]	35	48	15	55	256

**Table 4.** Properties of the PIN-PMN-PT single crystal and its 2-2 and 1-3 composites.

	***ρ* (kg/m^3^)**	${ɛ}_{33}^{T}$	***d*_33_ (pC/N)**	***k*_t_**	***Q*_m_**	***v* (m/s)**	***Z* (MRayl)**
**PIN-PMN-PT**	8000	4400	1510	0.57	120	4500	36
**2-2 composite**	5600	2902	1200	0.86	-	3856	21
**1-3 composite**	5420	2040	1256	0.84	6.8	3560	19

**Table 5.** Performance comparison of PIN-PMN-PT single crystal, 2-2 composite and 1-3 composite transducers.

**Transducer Type**	***f*_c_ (MHz)**	**BW (%)**	**IL (dB)**
Single-crystal ultrasonic transducer	2.06	70.9	25.4
2-2 composite ultrasonic transducer	2.11	93.1	21.3
1-3 composite ultrasonic transducer	1.95	94.6	21.1

**Table 6.** Temperature dependence of the PMN-PT and PIN-PMN-PT linear array performance [76].

**Linear Array**	**Temperature (°C)**	**f_c_ (MHz)**	**BW (%)**	**IL (dB)**
PMN-PT	21.5	3.36	57.14	27.65
	40	3.40	64.71	28.62
	60	3.29	61.80	29.51
	80	3.30	60.61	30.20
	100	3.53	71.95	31.04
	120	3.21	61.68	30.27
	140	3.24	53.09	33.40
	160	3.05	47.87	42.90

PIN-PMN-PT	21.5	4.10	72.53	36.31
	40	4.12	72.66	36.44
	60	3.89	65.64	35.97
	80	4.04	78.56	36.94
	100	3.91	66.33	34.83
	120	4.07	73.22	36.38
	140	4.11	72.51	37.24
	160	3.96	66.67	37.58

**Table 7.** Performance of each element for the annular array [88].

**Element**	**Center Frequency (MHz)**	**Bandwidth (%)**	**Insertion Loss (dB)**
1	35.3	41	21
2	33.3	48	18
3	35.4	46	27
4	35.0	46	25
5	35.8	55	26
6	30.0	78	28
7	36.8	67	24
8	33.3	57	26
